# Sedation in Critically Ill Children with Respiratory Failure

**DOI:** 10.3389/fped.2016.00089

**Published:** 2016-08-24

**Authors:** Nienke J. Vet, Niina Kleiber, Erwin Ista, Matthijs de Hoog, Saskia N. de Wildt

**Affiliations:** ^1^Intensive Care, Erasmus MC – Sophia Children’s Hospital, Rotterdam, Netherlands; ^2^Department of Pediatrics, Erasmus MC – Sophia Children’s Hospital, Rotterdam, Netherlands; ^3^Department of Pediatric Surgery, Erasmus MC – Sophia Children’s Hospital, Rotterdam, Netherlands; ^4^Department of Pediatrics, CHU Sainte-Justine, Montreal, QC, Canada; ^5^Department of Pharmacology and Toxicology, Radboud University, Nijmegen, Netherlands

**Keywords:** sedation, PICU, critically ill child, respiratory failure, pharmacokinetics, pharmacodynamics

## Abstract

This article discusses the rationale of sedation in respiratory failure, sedation goals, how to assess the need for sedation as well as effectiveness of interventions in critically ill children, with validated observational sedation scales. The drugs and non-pharmacological approaches used for optimal sedation in ventilated children are reviewed, and specifically the rationale for drug selection, including short- and long-term efficacy and safety aspects of the selected drugs. The specific pharmacokinetic and pharmacodynamic aspects of sedative drugs in the critically ill child and consequences for dosing are presented. Furthermore, we discuss different sedation strategies and their adverse events, such as iatrogenic withdrawal syndrome and delirium. These principles can guide clinicians in the choice of sedative drugs in pediatric respiratory failure.

## Introduction

Critically ill children who are mechanically ventilated often require sedative and/or analgesic drugs to diminish anxiety or pain and ensure comfort. Moreover, adequate sedation facilitates synchronization with mechanical ventilation and enables invasive procedures to be performed. Adequate sedation has been described as the level of sedation at which patients are asleep but easily arousable (1). In pediatric intensive care unit (PICU) practice, this means that a child is conscious, breathes in synergy with the ventilator, and is tolerant of or compliant with other therapeutic procedures. However, the optimal level of sedation varies for each patient, depending on the type and severity of underlying disease and the need for certain therapeutic, invasive procedures.

To achieve the optimal level of sedation in individual patients, doses of sedatives are preferably titrated to effect based on observational sedation scales validated for the population in question. Nonetheless, it can be difficult to reach optimal sedation because of variability in plasma drug levels and response as well as in the patient’s clinical state. Both under- and oversedation are undesirable, as these conditions may adversely affect patient outcomes. Oversedation delays recovery, as greater sedatives consumption is associated with longer duration of ventilation as well as extubation failure (2). Part of this effect may be due to muscle weakness consequent to immobility (3). Oversedation also induces tolerance and withdrawal syndrome (4, 5). Undersedation, on the other hand, may cause distress and adverse events such as unintentional extubation or displacement of catheters, may lead to adverse memories (posttraumatic-stress syndrome) and increased need for nursing requirements. All this may lead to a longer PICU stay.

This article addresses how to assess the need for sedation, including relevant sedation scales, pharmacokinetic (PK) and pharmacodynamic (PD) considerations of analgosedative drugs, sedation strategies, and long-term adverse effects of sedation, to guide clinicians to optimal sedation practice in pediatric respiratory failure. Moreover, we aim to elucidate the information gaps in current knowledge and propose future research directions.

## Sedation Assessment

In order to provide adequate sedation, the level of sedation in critically ill children should be regularly assessed and documented. Furthermore, sedation assessment is needed to both determine the efficacy of sedatives and related interventions and to facilitate inter-institutional comparisons. Thus, the use of formal sedation assessment is recommended using a validated sedation scoring scale. Several behavioral assessment tools are described. The Ramsay and the Richmond Agitation Sedation Scale (RASS) are frequently used in critically ill children, but are only validated for adult ICU patients (6–8). The COMFORT scale (9, 10), the COMFORT behavior scale (11, 12), and the State Behavioral Scale (SBS) (13) are validated scores for PICU patients. The characteristics and psychometric properties of these scales are presented in Table 1.

**Table 1:** **Characteristics of the COMFORT (behavior) scale and the State Behavior Scale**.

Instrument	Parameter measured	Population (age)	Exclusion criteria	Observation items	Score range	Validation		Cutoff points
					Item/total	Reliability	Validity	
COMFORT scale (9, 10)	Distress	37 (newborn to 17 years)	Seriously compromised neurological status, Profound mental retardation, Recent multiple trauma, Altered muscle ton or contractures, severe acute pain	Heart rate, mean arterial pressure, alertness, calmness, respiratory response, movement, muscle tone, facial expression	Numerical item: 1–5/total: 8–40	*r* = 0.84; *p* < 0.01 (*n* = 50 paired obs)	COMFORT vs. VAS *r* = 0.75; *p* < 0.01	OS ≤16
								AS 17–29
								US ≥30
COMFORT behavior scale (12)	Distress/sedation	78 (0–16 years)	Children with severe mental retardation, children with severe hypotonia, and patients receiving neuromuscular blockade	Alertness	Numerical item: 1–5/total: 6–30	Kappa = 0.77–1.0 (*n* = 40 paired obs)	COMFORT behavior vs. NISS (Kruskal–Wallis, *p* < 0.001)	OS ≤10
				Calmness/agitation				
				Respiratory response or crying				AS 11–22
				Physical movement		ICC = 0.99		
				Muscle tone				US ≥23
				Facial tension				
State Behavior Scale (13)	Sedation/agitation level	91 (6 weeks to 6 years)	Patients receiving neuromuscular blockade, postoperative patients, patients assessed to be in pain, unstable patients, patients at risk for opioid withdrawal	Respiratory drive, coughing, best response to stimuli, attentiveness to care provider, tolerance to care, consolability, movement after consoled	Bipolar numeric Item: −3 to +1/Total: −21 to 7	Kappa = 0.44–0.76 (*n* = 198 paired obs) ICC = 0.79	SBS vs. NRS (*F* = 75.8, *p* < 0.001)	Not done

*PICU, pediatric intensive care unit; VAS, visual analog scale; NISS, nurse interpretation sedation score; SBS, state behavior scale; NRS, numeric rating scale; kappa, linearly weighted Cohen’s kappa; r, Pearson product correlation coefficient; obs, observations; OS, Oversedation; AS, Adequate sedation; US, undersedation; ICC, intraclass correlation coefficient*.

The COMFORT scale was originally described in and validated for measuring discomfort in ventilated pediatric patients. This observational scale consists of two physiological items – heart rate and arterial blood pressure – and six behavioral items – alertness, calmness/agitation, respiratory response, physical movement, muscle tension, and facial tension. Because the physiologic variables are affected by inotropic and other drugs often used in pediatric intensive care, it was questioned whether their use contributes to the overall assessment of sedation in the individual patient. Therefore, the COMFORT scale was adapted in the COMFORT behavior scale, which does not include the two physiological items. Many psychometric properties of this scale have been tested (14–16). As well-sedated children do not always show unambiguous behavior, it was more realistic to define score ranges rather than cutoff points. Score range 6–10 was defined as oversedation; score range 23–30 as undersedation. Score range 11–22 was defined as a gray area in which a second assessment, for example the Nurse Interpretation of Sedation Score (NISS), is recommended for clinical purposes (12, 17).

The SBS appraises seven behavioral dimensions; “Respiratory drive/response to ventilation,” “Coughing,” “Best response to stimulation,” “Attentiveness to care provider,” “Tolerance to care,” “Consolability,” and “Movement after consoled.” The score range from −3 to +3 and a score of 0 describes a patient who is alert and calm. Psychometric properties of this scale are good.

## General Considerations of Pharmacokinetics and Pharmacodynamics in Critically Ill Children

**Figure 1:**
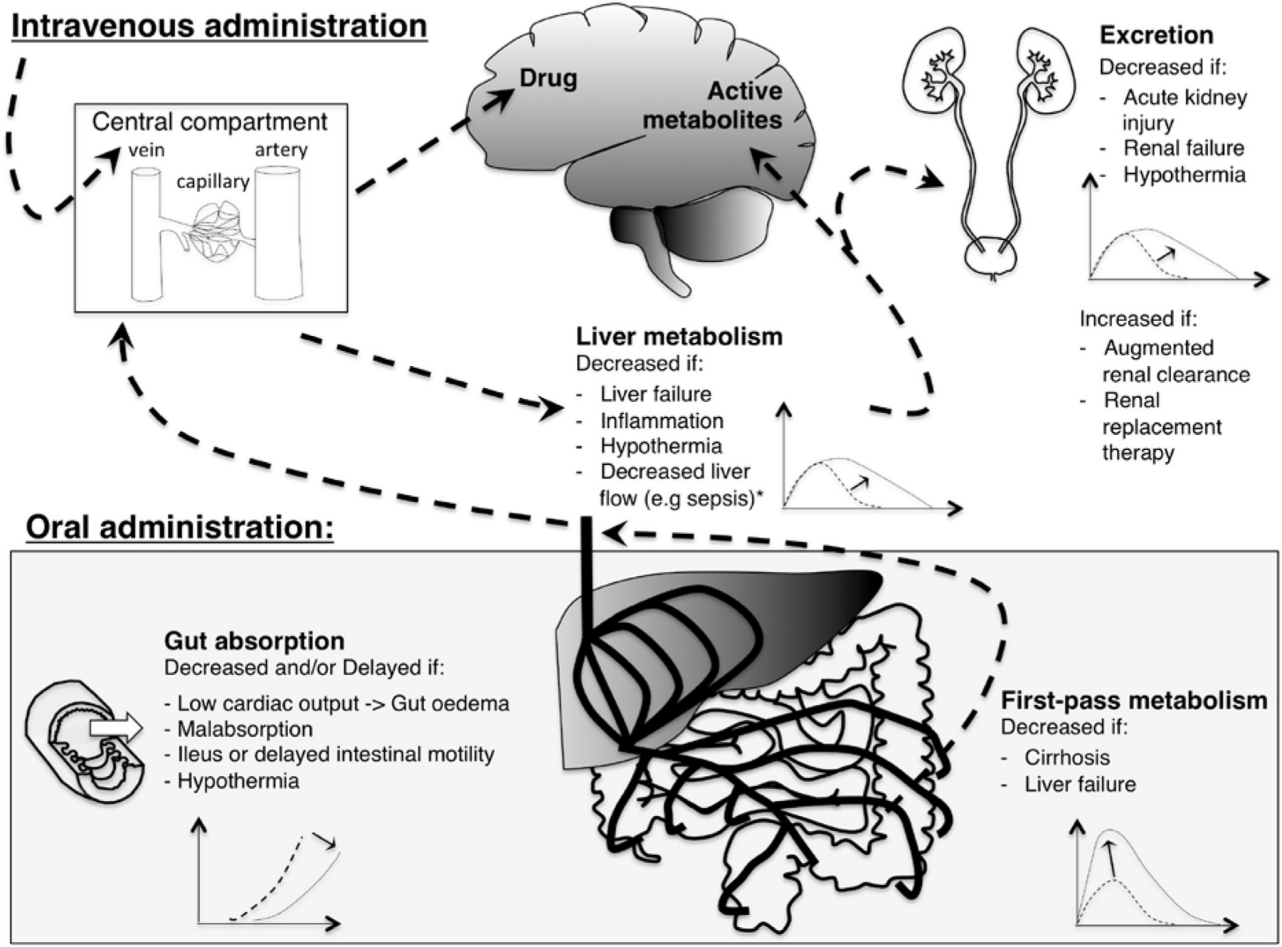
**Illustration of the effect of critical illness on pharmacokinetics of analgosedative drugs**. With intravenous administration (upper left), drugs are injected directly into the central compartment: bioavailability is complete. With oral administration (lower left), gut absorption and first by-pass metabolism limit bioavailability. Analgosedative drugs are metabolized by the liver into more water-soluble metabolites that are excreted by the kidneys. Some analgosedatives have active metabolites (e.g., morphine and midazolam) that may accumulate with decreased renal function. A graphical representation of drug concentration over time depicts pharmacokinetics changes induced by critical illness: the dashed line represents the curve of a healthy individual while the solid line shows the change induced by critical illness. *Liver flow affects clearance of drugs with a high hepatic extraction ratio (e.g., propofol).

The pharmacokinetic (PK) properties of a drug include the processes of absorption, distribution, metabolism, and excretion, while the pharmacodynamic (PD) properties comprise the actual responses to the administered drug and therefore may represent both efficacy and safety. In addition to the age-related variation in PK, critical illness and its treatment modalities impact PK and PD. These factors are summarized in Figure 1. Intrinsic factors related to the patient’s clinical condition include shifts in body fluid (altering volume of distribution), inflammation (altering drug transport and metabolism, clearance), and liver, renal, and heart failure (altering absorption, distribution, drug metabolism and excretion). Extrinsic factors include treatment modalities such as extra-corporeal membrane oxygenation (ECMO), hypothermia, and continuous renal replacement therapy (18). Volume of distribution is often increased and clearance is altered either way in ECMO patients (19). Hypothermia leads to changes in volume of distribution due to redistribution of blood flow and a decreased clearance due to a decreased drug metabolizing enzyme activity (20, 21).

Furthermore, critical illness itself may be of influence on the effect of sedation. For instance, a critically ill child who is less reactive due to its underlying illness (e.g., sepsis) will respond differently to a sedative drug than a relatively healthy child who receives sedation for the acceptance of a tube after airway reconstruction.

Although the impact of separate aspects of critical illness on drug disposition is increasingly recognized, only few factors are actually taken into account in current dosing such as dosing adjustments with renal failure. For sedative drugs, this underscores the importance of dosing and titrating the drugs to effect.

## Commonly Used Agents

An ideal sedative drug exhibits anxiolysis, amnesia, and analgesia qualities, should be easily titrated to effect, and without any adverse effects. However, none of the existing drugs does meet all these qualities. Therefore, medications are commonly co-administered to compensate for any shortcomings and to achieve an optimal effect.

In PICU, benzodiazepines and opioids are frequently used agents. Despite the widespread use of sedatives in PICU, high-quality data supporting appropriate dosing and safety are lacking (22). Many commonly used sedatives and analgesics in the PICU (e.g., lorazepam, dexmedetomidine, and fentanyl) are still used off-label, which means that their efficacy and safety have not been adequately proven (23). A rational choice for a particular agent is based on the desired effect of the drug, the interaction of the patient’s disease, and the side-effects of the drug (Table 2). These systemic effects can be adverse effects [e.g., propofol is avoided in patients with unstable hemodynamics due to its cardiodepressive properties (24)] or desired effects [ketamine is a bronchodilator used in asthma (25)]. Ideally, the choice for a particular agent should include its long-term effect on neurodevelopment. Most commonly used sedative and analgesics are neurotoxic in animals (26–28), which has caused uncertainty for their long-term safety in humans. Reassuringly, these animal data have not been confirmed in human studies. No adverse long-term effects of morphine administration at neonatal age were reported (29, 30). Moreover, short duration sevoflurane anesthesia in infancy does not appear to increase the risk of adverse neurodevelopmental outcome at 2 years of age compared with awake-regional anesthesia (31).

**Table 2:** **Drugs used for sedation in critically ill children and their PKPD considerations**.

	Indications	Dose	Elimination/metabolism	Effect of age on PK/PD	Dosing adjustment in organ impairment	
					Liver^a^	Renal
**Benzodiazepine**						
Midazolam	Sedation/amnesia	50–300 mcg/kg/h i.v.	Liver (CYP3A4/5) active metabolite: 1-OH-midazolam and 1-OH-midazolam glucuronide	CYP3A4/5 activity is low at birth and reaches adult values in the first years of life (106)	Consider (107)	Yes, in severe renal failure (108)
Lorazepam	Sedation/amnesia	0.01–0.1 mg/kg/h i.v.	Liver (glucuronidation by multiple UGT2B enzymes)	UGT2B7 low at birth and increases with age (109)	Consider (110)	No (108)
			No active metabolite			
**α-2 agonist**						
Dexmedetomidine	Sedation and analgesia	0.2–0.7 mcg/kg/h i.v.	Liver (glucuronidation and mainly CYP2A6)	Decreased clearance in children <1 years of age (111)	Yes	No
			No active metabolite			
Clonidine	Sedation and analgesia	0.5–2.5 mcg/kg/h i.v.	50% renal elimination/50% liver metabolism (mainly CYP2D6)	Decreased clearance in neonates	Consider	Yes/not significant
			No active metabolite			
**Propofol**	Sedation and hypnotic	1–4 mg/kg/U i.v. <24 h duration	Rapid and extensive liver metabolism (mainly CYP2B6)	Preterm neonates and neonates in the first week of life at increased risk for accumulation (112)	Consider (113, 114)	No
			No active metabolite			
**S-ketamine**	Analgesia and sedation	1–3 mg/kg/h (sedation)	Liver metabolism (demethylation and hydroxylation)	Appears similar to adults from 1 week onward (115)	Hepatotoxic (116)	No
			Active metabolite: norketamine (around three times less potent than ketamine)			
**Pentobarbital**	Sedation	1–5 mg/kg/h iv	Liver (microsomal enzyme system)	Reduced clearance in neonates (117)	Consider (118)	No (119)
			No active metabolite			
**Opioids**						
Morphine	Analgesia with sedation	5–40 mcg/kg/U i.v.	Liver (glucuronidation by UGT2B7)	Age-dependent increase in plasma clearance in children younger than 10 years of age (109)	Consider (120)	Initiate at lower dose and titrate slowly (121)
			Active metabolite: morphine-6-glucuronide (more potent than morphine)			
Fentanyl	Analgesia and sedation	1–10 mcg/kg/h iv	Liver (CYP3A4)	NA	Consider	Yes
**Benzodiazepine**						
Midazolam	Increased Vd and drug loss *in vitro* (122, 123)	Hypotension with bolus dosing (124)	Respiratory depression	++ (5, 83)		
		Fall in cardiac output (125)				
Lorazepam	High drug loss *in vitro* (126)	Hypotension	Respiratory depression	++ (83)		
**α-2 agonists**						
Dexmedetomidine	No data	Bradycardia and hypotension rarely of clinical significance	No significant respiratory depression, useful for extubation of in spontaneously breathing patient	Rebound hypertension and possible withdrawal after prolonged infusion (weaning required or switch to oral clonidine) (127)		
Clonidine	No data	Bradyarrhytmia has been reported		Rebound hypertension and withdrawal (weaning required)		
**Propofol**	High drug loss *in vitro* (19, 128)	Myocardial depressant	Respiratory depression	Irritability, jitteriness, and agitation on abrupt discontinuation after prolonged infusion (100)		
	No *in vivo* study		Very quick emergence by stopping, useful during weaning of mechanical ventilation			
**Ketamine**	No data	Usually preserved hemodynamic stability, but when endogenous stores of catecholamines have been depleted by stress or chronic illness ketamine can induce cardiovascular depression.	No respiratory depression	Delirium after prolonged use in adult		
			First-line sedative in asthma (Bronchodilator)	No data in PICU		
**Barbiturate**	Increased Vd (129)	Hypotension, depression of cardiac contractility	Respiratory depression	++ (73)		
**Opioids**						
Morphine	High drug loss *in vitro* (126, 130)	Histamine release leading to vasodilatation and hypotension, particularly following bolus dose	Respiratory depression	++ (83)		
	Clearance and Vd changes during prolonged ECMO (131)		Use with caution in asthmatic patients due to potential histamine release			
Fentanyl	High drug loss *in vitro* (123, 130)	Large bolus doses can cause hypotension	Respiratory depression	++ (83)		

*^a^All drugs that are significantly metabolized by the liver may need adjustment in fulminant acute liver failure, but not with mild increases of liver enzymes. Consider using only bolus doses and titrate to effect or use non-hepatically cleared drug like remifentanil*.

### Benzodiazepines

Benzodiazepines (midazolam, and to a lesser extent lorazepam) are the most commonly used sedatives and the sedative of choice in many pediatric intensive cares (32). Midazolam is a central nervous system depressant that exerts its clinical effect by binding to a receptor complex, which facilitates the action of the inhibitory neurotransmitter gamma-amino butyric acid (GABA) in the brain. Through this effect, midazolam possesses sedative, anxiolytic, anticonvulsant, muscle relaxant, and amnesic properties (33). The amnesic effects of midazolam probably play an important role in the low levels of unpleasant experiences recalled by survivors of PICU treated with this agent (34).

Midazolam is metabolized to a major hydroxylated active metabolite (1-OH midazolam) by CYP3A4/5, and subsequently metabolized to 1-OH-midazolam–glucuronide by UGTs and renally excreted (35). A reduction of CYP3A activity as a result of inflammation, organ failure (36), or concomitant administration of other therapeutic drugs (drug–drug interactions) (37) may account for the failure of critically ill children to metabolize midazolam. In patients with renal failure, prolonged sedative effects may be caused by the accumulation of the active metabolite, 1-OH midazolam–glucuronide (38).

Although most commonly used, midazolam is certainly not an ideal sedative agent. Adverse events associated with its use are not only tolerance, dependence, and withdrawal but also paradoxical hyperactivity (4, 5). In adults, continuous benzodiazepine use is associated with prolonged mechanical ventilation and length of ICU stay (39). Also, hypotension may occur and is most likely with bolus administration, particularly in neonates, in the setting of hypovolemia or concomitant use of morphine (40).

Lorazepam is a long-acting benzodiazepine used orally and intravenously. The use of intravenous lorazepam is limited by the fact that it is dissolved in propylene glycol, which can accumulate to produce metabolic acidosis and renal dysfunction (41, 42). For weaning, oral lorazepam is a good alternative for midazolam, because of its long half-live.

### Opioids

Although opioids are analgesic drugs, they have sedative effects. Some PICUs use morphine as a first-line sedative while others favor sedatives (mainly benzodiazepine) in the absence of suspected pain (43). Morphine provides sedation as well as analgesia and can be used as a single agent for analgesia and sedation.

As morphine clearance is substantially reduced in neonates less than 10 days of age, 1/3–1/2 of dosing in older children is needed to reach the same plasma levels as in older children. For analgesia, this dose reduction is related to adequate analgesia, but sedation data are lacking (44). Morphine has a relatively long duration of action of around 2 h when administered as a single dose intravenously (peak analgesic effect after 20 min). Morphine is characterized by hepatic metabolism (glucuronidation) and renal excretion with intermediate volume of distribution. Therefore, its effects can be prolonged in patients with renal impairment. The impact of liver failure seems mild or moderate at best (45). Morphine stimulates the release of histamine and inhibits compensatory sympathetic responses, leading to vasodilation and consequently hypotension, particularly following bolus administration (46). The opioid fentanyl has powerful analgesic properties and provides some sedation, as demonstrated in a randomized controlled trial comparing continuous fentanyl and remifentanil in postoperative orthopedic children (47). No studies are available for the use of fentanyl for long-term sedation in PICU. An important, but rare adverse effect is fentanyl-induced chest wall rigidity causing respiratory compromise, generally occurring after a large fentanyl bolus administration (48).

### Alpha-Agonists

Clonidine and dexmedetomidine are central α-2 agonists with sedative and analgesic properties (49) increasingly used as first-line sedatives or as adjunct to other sedatives. Enthusiasm for these agents is driven by the absence of clinically significant respiratory depression (49, 50), which is an advantage in the spontaneously breathing patient or when extubation is planned (51). Moreover, they do not show neurotoxicity in animals (52), have opioid and benzodiazepine sparing properties (53, 54), and may decrease the incidence of withdrawal and delirium (55). A RCT comparing continuous intravenous clonidine and midazolam in 129 ventilated children (30 days to 15 years) showed a similar sedative effect (56). Sedation under dexmedetomidine may more closely resemble natural sleep than sedation under benzodiazepines, although these theoretical advantages have not yet been demonstrated to improve patients’ perception of sleep in adult ICU (57, 58). For children, the use of dexmedetomidine is still off-label; it is approved for continuous sedative infusion in adults for 24 h.

The main adverse effect of alpha agonists is bradycardia/arythmia and hypotension (49, 59), but these effects are rarely of clinical significance (54, 56). Data in children with severe hemodynamic compromise are insufficient to recommend their use in this particular population. To date, no study compared dexmedetomidine to clonidine in the PICU.

### Propofol

Propofol has sedative and hypnotic properties. It involves GABA receptor activation (60) although its mechanism of action is not fully understood. Due to its strong cardio-depressant effect (24), its use should be avoided in the hemodynamically unstable patient. Long-term infusions in the PICU are contraindicated in the official drug label for children <16 years due the risk of lethal propofol infusion syndrome (PRIS). Any suspicion of PRIS (clinical and biological signs: metabolic acidosis, increased liver enzymes, lipemia, rhabdomyolysis, renal and cardiac failure) should lead to an immediate interruption of propofol infusion but despite discontinuation, death can ensue (61). Propofol infusion rate and duration, the presence of traumatic brain injury, and fever are factors associated with mortality in PRIS (62). The use of propofol should be limited; when used maximum infusion rate must not exceed 4 mg/kg/h with a maximum duration of 24 h (62).

Propofol’s very short half-live offers an advantage around the time of extubation (mainly in agitated patients): it allows weaning from the longer acting sedative inducing respiratory depression, control sedation during the time of extubation and ensure a quick recovery after. Therefore, in this special case, a short-term infusion of propofol can be considered.

### Ketamine

Ketamine is an NMDA receptor antagonist (63) with cataleptic, amnestic, and analgesic properties. It maintains hemodynamics (64, 65) by inducing release of endogenous catecholamine (65). However, in patients with hemodynamic compromise and chronic illness or stress who have depleted catecholamine stores, it can decrease myocardial contractility and even induce collapse (66, 67). Ketamine is used in the PICU as a co-analgesic with opioids for pain control (low dose, around 0.1 mg/kg/h) (68) and occasionally when usual sedative agents fail to provide adequate sedation (high dose, 1–3 mg/kg/h). Due to its bronchodilatory properties, it is the first-line analgosedative in status asthmaticus (25, 69). A very common adverse effect of ketamine is the occurrence of hallucinations, and therefore low dose of benzodiazepines should be co-administered. Early work hints at its potential to elevate intracranial pressure (70) and many physicians still avoid its use in traumatic brain injury despite more recent work not showing this effect (71).

### Antihistamines

Promethazine, alimemazine, and diphenhydramine are first generation antihistamines with anti-dopaminergic and anticholinergic drug actions. These drugs may produce significant sedation as well as quiescence. A combination of oral chloral hydrate and promethazine was more effective than midazolam infusion for maintenance sedation in critically ill children, but less than half the patients in each study arm reached target sedation during study period (72). No other studies are available, and therefore, evidence to use antihistamines for (long-term) sedation in PICU is low.

### Barbiturates

Pentobarbital and thiopental are primarily used for therapy-resistant status epilepticus, but its use as sedative in therapy-resistant agitation has also been reported (73, 74). Barbiturates are highly lipid soluble. Given by infusion, it accumulates in adipose tissue whence it diffuses slowly back to the blood after infusion cessation. This, coupled with a long half-life (5–10 h), is responsible for the persistence of sedation after infusion cessation. Barbiturates are also associated with high rates of adverse events, including hypotension, depression of cardiac contractility, severe skin and mucous reactions (Stevens Johnson syndrome and Toxic Epidermal Necrolysis), and neurologic sequelae (73). Life-threatening hypokalemia and rebound hyperkalemia have been observed after cessation of thiopentone coma for intracranial hypertension. As this has not been observed with other underlying diseases or with pentobarbital, its cause is likely due to an association between the underlying clinical symptoms and thiopentone (75).

### Neuro-Muscular Blockers

Analgesia and profound sedation have to be ensured before starting neuromuscular blockade. Neuromuscular blocking agents are associated to critical illness polyneuropathy and myopathy and therefore should be restricted to special circumstances, discontinued as soon as possible and used at the smallest possible dose (76, 77). The level of evidence supporting their prolonged use for particular indications is poor (76, 78). They are recommended if effective mechanical ventilation cannot be achieved despite profound sedation [e.g., ARDS (77), severe asthma (25, 79)]. They are often used in case of severe cardio-vascular instability, but their benefit may be limited because only modest decrease in energy consumption is achieved compared to profound sedation (80, 81). Other common uses are refractory pulmonary and intracranial hypertension (82).

## Sedation Strategies

Optimizing sedation in the critically ill is of major importance. In general, the current tendency is to lighten sedation in the intensive care to avoid delayed recovery with longer duration of ventilation (2), tolerance, and withdrawal (5, 83). Despite the awareness of the adverse effects of oversedation, it remains common practice in the PICU (84). Sedation strategies play a key role to achieve adequate sedation (Box 1).

Box 1 **Practical recommendations for physicians.**

Step 1. Assessment

– Use a validated sedation scale and train all nurses to adequately use this scale.
– Assess the level of sedation in critically ill children regularly (e.g., COMFORT-b scale every 8 h and additionally in case of distress and after interventions).
– Identify the desired level of sedation for the individual patient and act when over- or undersedated.

Step 2. Non-pharmacological treatment

– Reduce distress by nursing and parenting interventions.

Step 3. Pharmacological treatment

– Titrate the sedatives to achieve the optimal level of sedation for that individual patient.
– Start with one drug, choice preferably protocolized: e.g., midazolam, lorazepam, morpine, or fentanyl and titrate up.
– If distress, add one drug from other class, e.g., opioid when already benzodiazepine.
– If sedation is still insufficient with these two drugs, add clonidine, dexmedetomidine, or ketamine, consider switching benzodiazepine or opioid.
– Always give a bolus dose with increase of infusion, to quickly reach steady-state unless the patient is hemodynamically too unstable, than consider a bolus dose in 30 min.
– If all sedatives fail, consider pentobarbital and discontinue other drugs. Be careful with abrupt discontinuation of α2-agonists (rebound hypertension) and opioids.

Step 4. Weaning and delirium

– Decrease sedatives based on sedation scores.
– Add withdrawal and delirium score at regular intervals.
– If a patient received sedatives >5 days, consider slow tapering or switch to long-acting oral drugs.
– When scores suggest delirium, consult psychiatrist for diagnosis.
– When antipsychotics are considered necessary: start low, go slow, monitor adverse events.

### Protocolized Sedation

To optimize sedation in critically ill children, it is recommended to assess levels of sedation and to titrate sedatives and analgesics on the guidance of sedation protocols or algorithms. Implementing a sedation protocol allows targeting patient-specific sedation goals. In the adult intensive care, protocol implementation decreases days of mechanical ventilation and ICU stay (85). But more recently, adult studies failed to show these positive effects (86). These changes in results over time may be explained by the growing awareness of the deleterious effect of oversedation and general tendency to avoid it. In the PICU, the effect of protocolizing sedation is less clear, but studies are recent and avoidance of oversedation may already have entered the practice. Several non-randomized trials reported conflicting results on the impact of protocolized sedation on outcomes like length of PICU stay, duration of mechanical ventilation, or the need for analgesia and sedation (87). Recently, in a large cluster randomized trial among children undergoing mechanical ventilation for acute respiratory failure, the use of a sedation protocol compared to usual sedation practice did not improve clinical outcome (88).

### Daily Sedation Interruption

Another approach to potentially avoid the negative effects of oversedation, and especially the adverse effects of continuous benzodiazepine use, is daily sedation interruption (DSI). In adults, clinical trials have shown that DSI can reduce the duration of mechanical ventilation, hospital stay, and amount of sedatives administered, without compromising patient comfort or safety (89). Several later studies have confirmed this beneficial effect (90), whereas other studies, in different settings, showed no benefit (91, 92).

In critically ill children, two pilot studies showed that DSI is feasible and safe, even in ECMO patients, but both studies were not designed to detect differences in clinical outcome (93, 94). Another study, comparing DSI with continuous sedation in children, DSI led to improved clinical outcomes, including shorter durations of mechanical ventilation and PICU stay (95). In a recent study comparing DSI + protocolized sedation to protocolized sedation only, no beneficial effect of DSI was found (96). DSI did not reduce the duration of mechanical ventilation, length of stay, or the amounts of sedative drugs administered. There are important differences between these studies in study design (DSI and Standard of Care arm vs. DSI + protocolized sedation and protocolized sedation arm), setting (India vs. Europe), patient population (e.g., high incidence of neurotrauma vs. respiratory infection), and ICU practices (e.g., longer mean duration of mechanical ventilation, more sedatives and neuromuscular blockers administered in the first study) (97). For the latter study (DSI + PS vs. PS), the effect of protocolized sedation itself on the clinical endpoints might have outweighed the effect of DSI, as also demonstrated in adults (91).

### Drug Cycling

Some PICUs use drug “cycling” or “rotation” as a method of decreasing the adverse effects of continuous sedation (98). This strategy is aimed at preventing tachyphylaxis and tolerance by “cycling” drug combinations. For example, an opioid and benzodiazepine regimen can be changed to ketamine and promethazine, followed by clonidine and chloral hydrate, all on a weekly basis. However, to the best of our knowledge, evidence supporting the beneficial effects of “cycling” is lacking.

## Adverse Effects

### Withdrawal

Prolonged administration of analgesics and sedatives in critically ill children may induce drug tolerance and physical dependency. Abrupt discontinuation or too rapid weaning of these drugs in physically dependent children may cause withdrawal syndrome. Symptoms of benzodiazepines and opiates withdrawal can broadly be distinguished into three groups: (1) overstimulation of the central nervous system (e.g., agitation, tremors, anxiety, and hallucinations), (2) autonomous dysregulation (e.g., sweating, fever, tachycardia, and tachypnea), and (3) gastro-intestinal symptoms, which have only been described in opiate withdrawal (99). Withdrawal syndrome has been particularly reported after administration of opioids and benzodiazepines. The onset of withdrawal syndrome depends on the half-life of the drug and can be after 1 h or up to several days after discontinuation of these drugs (100). Both longer duration of administration and high total doses of opioids and/or benzodiazepines are clearly related with the occurrence of withdrawal syndrome in critically ill children, and may therefore be considered risk factors (83, 99). Moreover, the exact biochemical mechanisms responsible for the development of withdrawal syndrome remain unclear. The reported prevalence of withdrawal syndrome in critically ill children who had received benzodiazepines and/or opioids for 5 or more days ranges from 17 to 57% (99, 101).

The development of pediatric scoring tools for withdrawal syndrome is a huge step forward. Two validated assessment tools for observing and identifying withdrawal syndrome after long-term use of benzodiazepines and opioids in PICU patients have been described. These are the Withdrawal Assessment Tool version-1 (WAT-1) and the Sophia Observation Withdrawal Symptoms-scale (SOS) (102–105). Table 3 provides details on symptoms and psychometric properties of the WAT-1 and SOS. The WAT-1 is an 11-item scale and scores of three or higher (on a scale of 0–12) which indicates that the child is suspected for withdrawal. The SOS consists of 15 items and is based on the underlying empirical structure of co-occurrences of withdrawal symptoms that experts considered relevant. A SOS score of 4 or higher reflect a high probability of withdrawal.

**Table 3:** **Symptoms and psychometric properties of the WAT-1 and SOS**.

Instrument	Population	Observation items	Structure		Psychometric evaluation		Withdrawal cut-off scores
			Total items	Score-range	Reliability	Validity	
Withdrawal Assessment Tool version 1 (WAT-1) (102, 103)	Children	Tremor	11 Numerical	0–12	*Internal*: PRINCALS, 4 factors	*Construct*: Sen. = 0.87, Spec. = 0.88	≥3
		Uncoordinated/repetitive movement					
						r_s_: 0.80 (between WAT-1 score and NRS-withdrawal)	
					*IRR*		
					*N* = 30 paired observations		
		Yawning or sneezing					
						Peak WAT-1 scores for each subject correlated moderately with total cumulative opioid exposure (*r* = 0.23, *p* = 0.009), cumulative benzodiazepine preweaning (*r* = 0.30, *p* < 0.001) and total (*r* = 0.33, *p* < 0.001) exposure	
		State					
					ICC = 0.98		
					Cohen’s kappa = 0.80		
		Loose/watery stools					
		Vomiting/retching/gagging					
		Temperature >37.8°C					
						*Sensitivity to change*	
						*N* = 51 episodes of withdrawal (in 21 pts) WAT-1 score	
		Sweating					
						– before rescue therapy: 6 (4–8) – after after rescue therapy: 2 (1–3) (Wilcoxon–signed rank test *p* < 0.001)	
		State					
		Startle to touch					
		Time to gain calm state (SBS ≤ 0)					
Sophia Observation withdrawal Symptoms-scale (SOS) (104, 105)	Children	Tachycardia, tachypnea, fever (≥38.5°), sweating, agitation, anxiety, tremors, increased muscle tone, inconsolable crying, grimacing, sleeplessness, motor disturbance, hallucinations, vomiting, and diarrhea	15 Numerical	0–15	*Internal*: MDS, 3 dimensions	*Construct*	≥4
						85 experts	
					*IRR: N* = 23 paired observations, ICC = 0.97	*Construct*	
						Sen. = 0.83	
						Spec. = 0.93	
						*r*_s_: 0.51 95% CI 0.32–0.66, *p* < 0.001) cumulative doses of benzodiazepines	
					Cohen’s kappa = 0.73–1.0 (items)		
						*r*_s_: 0.39 (95% CI 0.17–0.57, *p* < 0.01) cumulative doses of opioids	
						*Sensitivity to change*	
						*N* = 156 paired SOS assessments in 51 pts	
						Decrease SOS score: 1.47 (95% CI, −1.91 to −1.04) after rescue therapy	

Strategies to reduce the prevalence of withdrawal syndrome should begin by making active efforts to reduce doses of benzodiazepines and/or opioids during the whole ICU course, and thereby preventing oversedation. As discussed above, DSI does not appear to add to protocolized sedation to reach this goal. Protocolized sedation targeting at conscious sedation appears at this time the best available approach.

A weaning strategy for gradual decreasing of opioid and/or benzodiazepine dosages once the patient is recovering may be effective to prevent withdrawal syndrome. Strategies include slowly tapering off the intravenous infusion rate over time, using an alternative route, e.g., enteral or subcutaneous, or transition to long acting drugs like methadone from morphine/fentanyl or lorazepam from midazolam. Disappointingly, little evidence is available on efficacy or safety of different weaning strategies. Weaning strategies ranging from 10 days to several months have been evaluated in observational (retrospective and prospective) studies (132–136). Two negative RCTs evaluated methadone weaning in 5 vs. 10 days (137) and a high- vs. low-dose methadone schedule in children (138). And while target drug levels for sedative and opioid dependence have been established for adults, they are lacking for children, as are PK data. Hence, we can not advise on the optimal weaning strategy or preferred drugs in pediatric ICU withdrawal.

Nevertheless, some suggestions to reduce withdrawal syndrome while avoiding unnecessary prolonged drug use can be made. First, awareness among clinicians on the risk factors for withdrawal symptoms may aid to prevent a too rapid reduction in drug doses. Moreover, it may lead to a faster switch from IV, short half life drugs to oral or subcutaneous, long half-life drugs. This may also facilitate faster ICU discharge. Second, regular monitoring of withdrawal symptoms with validated scales will also help to faster diagnose and treat withdrawal as well monitoring of the effect of interventions.

### Pediatric Delirium

Pediatric intensive care staff has become more alert to the occurrence of delirium in their patients – not least since studies showed an estimated incidence of 4–29% (139–141). The core diagnostic criteria for delirium are (a) disturbance of consciousness with reduced ability to focus, shift or maintain attention; (b) change in cognition (such as memory deficit, disorientation, language disturbance) or development of a perceptual disturbance; (c) the disturbance develops over a short period of time and tends to fluctuate during the course of the day. The pathogenesis of delirium is largely unknown. The sufferers may be hyperactive, hypoactive, or show signs of both states. Typical for the hypoactive delirium are slowed or sparse speech, hypoactive or slowed motor activity as well as lethargy or also described as reduced awareness or apathy. A number of delirium symptoms overlap with those observed in other conditions, such as pain and withdrawal syndrome (99).

Adults and children largely show the same symptoms although hallucinations, cognitive changes and hypoactive delirium are difficult to diagnose in the very young, preverbal PICU population. For this reason, PD is underdiagnosed in this age group (139, 141). Another reason is that nurses and physicians may not specifically focus on the symptoms of PD. Still, it is also possible to assess PD in this vulnerable age group by carefully observing behavior (139, 142, 143). Diagnosing of PD in the PICU setting requires a reliable, validated, and clinically useful bedside tool that may also serve for screening and guiding of treatment. This is an area in full development but several suitable instruments are already available: the pediatric Confusion Assessment Method for ICU (pCAM-ICU) (141), the Cornwell Assessment Pediatric Delirium tool (CAP-D) (140, 144), and the Sophia Observation withdrawal Symptoms-Pediatric Delirium scale (SOS-PD) (145). Haloperidol and risperidone are antipsychotics used for delirium in critically ill children and also adults. To date, studies showing benefit of antipsychotics to prevent or treat ICU delirium are lacking. Moreover, in a retrospective cohort of critically ill children, almost 10% of children showed severe adverse events associated with haloperidol treatment, including extrapyramidal syndrome (146). Hence, while ICU delirium has been associated with an increased risk of mortality, it is unclear if the benefits of antipsychotic treatment outweigh the risks. In the Netherlands, the Dutch Pediatric Drug Handbook (www.kinderformularium.nl) advices a low haloperidol starting dose to be carefully titrated to effect, while diligently monitoring potential side effects.

## Non-Pharmacological Approach

Drug therapy is the most obvious treatment modality of distress, withdrawal syndrome and delirium in critically ill children. Increasingly, the importance of non-pharmacological interventions is recognized. Such interventions use a multi-component approach, which including repeated reorientation, early mobilization, noise reduction (use of ear plugs), and a non-pharmacological sleep management. We suppose that these interventions could reduce distress and delirium, but evidence is limited. However, common sense suggests that these interventions (for example, promoting orientation and day-night rhythm, and avoiding overstimulation by light and sounds) may be effective for children as well.

Another strategy is adaptation of the environment, like noise reduction. Noise is a major environmental factor to cause anxiety and sleep disturbance in critically ill patients (32). In a way, noise reduction could well be effective in decreasing anxiety. It would be worthwhile, therefore, to reduce noise in the PICU as much as possible. All in all, based on the limited evidence it is difficult to extrapolate the effectiveness from adults to children. However, common sense has it that most of the interventions, for example promoting orientation and day–night rhythm and avoiding overstimulation by light and sounds, may be suitable for children as well, so as to create a comfortably calm environment for child and parents. Adult data show a reduction in delirium rates with a multifaceted approach, not only including lighter sedation approaches, but also non-pharmacological changes as noise reduction and aids for patients to better orientate themselves (147).

## Future Research

Despite the widespread use of sedatives to facilitate mechanical ventilation in pediatric intensive care, evidence to guide clinical practice is remarkably scarce. Only few adequately powered, well-designed RCTs to study efficacy and safety of individual drugs or their combinations have been performed. Several roadblocks to the conduct of these trails have been identified and should be taken into account with the design of future studies. Hence, robust study design including adequate power calculation, randomization procedures and blinding. (International) multi-center design is very likely needed to reach adequate sample size and high likelihood of generalizability. This adds complexity to the trial and asks a tremendous effort in training of local nurses, physicians and other study personnel. Validated sedation scales for the specific population, e.g., also taking into account age of patient and patient-controlled or nurse-controlled, must be used to assess sedation level in children.

Further, especially in critically ill children, “gate keeping,” i.e., not including the sickest patients for fear of overburdening patients and parents, presents an important challenge toward adequate recruitment. But, previous studies have shown that these challenges can be overcome and taking them into account, future research could focus on the following aspects of pediatric sedation in the ICU:

– Does protocolized sedation indeed improve clinical outcome? Preferably, short-term outcomes like as ventilator-free days, extubation readiness, withdrawal syndrome and long-term outcomes, like neurodevelopmental outcome, occurrence of PTSD and quality of life should be evaluated. This should also be evaluated in RCTs aiming to study non-pharmacological and pharmacological interventions.
– What are optimal drug doses to be used in pharmacological trials? Can we target similar drug concentrations in all patients, or do different patients need different target concentrations, e.g., based on severity of disease, underlying disease? Before a RCT can start, PK data should be available, from the literature or from prospective observational studies to explore PK and PD of the future study drugs. Especially, data is missing to guide dosing during critical illness and associated treatment modalities (e.g., CVVH and ECMO).
– Using a good understanding of the drug’s PK and preferably target concentration, these data should be used to design RCT’s comparing sedation regimens. Ideally, the PKs of the sedative drugs are also studied in these trials to validate the dosing assumptions and better understand variability in response.
– Another underrated aspect of drug trials is the recording of adverse events. A prospective, well-designed approach to document adverse events, may also aid to balance efficacy and safety of the different sedation approaches and guide future treatment decisions.
– Industry-initiated trials follow strict regulatory guidelines for the performance of clinical trials, including adequate documentation of adverse events, according to good clinical practice guidelines with extensive monitoring. Traditionally, these have been weaker in investigator-initiated trials, due to a lack of oversight and funds. Hence, consulting with experts in regulatory drug trials is important to safe-guard the quality and thereby also the safety of participants, as well as the generalizability of the results.

## Author Contributions

All authors contributed to the draft and critical revision of the manuscript. The study was supervised by de Wildt.

## Conflict of Interest Statement

The authors declare that the research was conducted in the absence of any commercial or financial relationships that could be construed as a potential conflict of interest. The reviewer GH and handling editor declared their shared affiliation, and the handling editor states that the process nevertheless met the standards of a fair and objective review.
